# Capacity of mosquitoes to transmit malaria depends on larval environment

**DOI:** 10.1186/s13071-014-0593-4

**Published:** 2014-12-14

**Authors:** Lillian L Moller-Jacobs, Courtney C Murdock, Matthew B Thomas

**Affiliations:** Center for Infectious Disease Dynamics and Department of Entomology, Merkle Lab, Pennsylvania State University, Orchard Road, University Park, PA 16802 USA; College of Veterinary Medicine, Odum School of Ecology, University of Georgia, DW Brooks Drive, Athens, GA 30602 USA

**Keywords:** *Anopheles stephensi*, Disease ecology, Food stress, Host-parasite interactions, Nutrition, *Plasmodium yoelii yoelii*, Trans-stadial effects

## Abstract

**Background:**

Adult traits of holometabolous insects such as reproduction and survival can be shaped by conditions experienced during larval development. These “carry-over” effects influence not only individual life history and fitness, but can also impact interactions between insect hosts and parasites. Despite this, the implications of larval conditions for the transmission of human, wildlife and plant diseases that are vectored by insects remain poorly understood.

**Methods:**

We used *Anopheles stephensi* mosquitoes and the rodent malaria, *Plasmodium yoelii yoelii*, to investigate whether quality of larval habitat influenced vectorial capacity of adult mosquitoes. Larvae were reared under two dietary conditions; one group received a diet commonly used for colony maintenance (0.3 mg/individual/day of Tetrafin fish food) while the other group received a reduced food diet (0.1 mg/individual/day). Upon emergence, adults were provided an infectious blood feed. We assessed the effects of diet on a range of larval and adult traits including larval development times and survival, number of emerging adults, adult body size and survival, gonotrophic cycle length, and mating success. We also estimated the effects of larval diet on parasite infection rates and growth kinetics within the adult mosquitoes.

**Results:**

Larval dietary regime affected larval survival and development, as well as size, reproductive success and survival of adult mosquitoes. Larval diet also affected the intensity of initial *Plasmodium* infection (oocyst stage) and parasite replication, but without differences in overall infection prevalence at either the oocyst or sporozoite stage.

**Conclusions:**

Together, the combined effects led to a relative reduction in vectorial capacity (a measure of the transmission potential of a mosquito population) in the low food treatment of 70%. This study highlights the need to consider environmental variation at the larval stages to better understand transmission dynamics and control of vector-borne diseases.

## Background

Malaria is the most important vector-borne disease of humans worldwide, with approximately 219 million people infected annually, resulting in about 600,000 deaths per year [1]. The transmission intensity of malaria is inextricably linked to the biology of the mosquito vectors and can be characterized using a summary metric known as the vectorial capacity (*C*). Vectorial capacity describes the rate at which future infections arise from a currently infected host (provided that all female mosquitoes become infected) and provides a measure of the transmission potential of a vector population [2,3]. It is defined as:

$$ C=\frac{m{a}^2b{p}^n}{- ln(p)} $$

where *m* is vector density (ratio of adult mosquitoes to humans), *a* is the daily probability of a human host being fed on by a vector, *n* is the extrinsic incubation period of the parasite, *p* is the daily probability of adult vector survival, and *b* is the proportion of mosquitoes with sporozoites disseminated in their salivary glands.

Any variation in environment that affects relevant aspects of vector biology could result in a change in transmission risk via effects on vectorial capacity [4-8]. Recent work shows that changes in temperature (both means and diurnal fluctuation) and rainfall events can have substantial effects on the transmission potential of malaria [7,9-11]. Other sources of environmental heterogeneity include differences in food resource availability, seasonality of habitats, and land use changes [12,13].

To date, many studies examining the effect of environment on mosquito biology and aspects of vectorial capacity have focused directly on the adult mosquitoes. This is logical, as it is only the adult female mosquitoes that transmit malaria and the frontline interventions used for control (such as insecticide treated bed nets, indoor insecticide sprays, screening, repellents etc.) primarily target the adult stage. Small changes in daily survival probability (*p*) and human biting rate (*a*), for example, can have very large effects on vectorial capacity, which explains in-part the effectiveness of insecticide treated nets as these act on both traits simultaneously [14-18]. Biting rate (determined by the duration of the gonotrophic cycle) has also been shown to be a major factor explaining variation in malaria incidence [18-20]. However, larval condition has become increasingly recognized as having an influence on adult mosquito life history traits [14-16,19-21]. Based on studies in other invertebrate systems, it is expected that variation in quality of larval habitats could feed through to impact adult life history, which in turn could affect transmission [22-24]. For terrestrial insects and other small invertebrates, estimates of body condition are often positively correlated with body size [25,26], and larger individuals often exhibit increased probability of survival, fecundity and ultimate overall fitness [27,28]. Adult survival and vector density are key elements of vectorial capacity. Larval effects on adult body size might also be important in determining vector competence. Larger individuals could support more parasites due to greater availability of host resources [29,30]. Alternatively, individuals with more reserves might be able to devote more energy toward immune defense [19,31].

Extensive studies on *Aedes* species have shown that larval environment can have considerable effects on life history traits important to transmission, such as development rate, adult longevity, and efficiency of egg development [32-34]. Larval environment has also been demonstrated to significantly shape vector competence in a variety of *Aedes*-arbovirus systems. For example, interactions between larval competition and density in *Aedes aegypti* and *Aedes albopictus* can significantly increase susceptibility to dengue virus and Sindbis virus [35,36]. Nutritional stress in *Aedes aegypti* has also been shown to influence the interaction between humoral and cellular branches of the immune system, which could affect vector competence for a suite of pathogens [37].

A more limited number of studies on *Anopheles* vector spp. support the influence of larval condition on subsequent adult traits and vectorial capacity, but the patterns are not well understood [15,38-40]. Here, we use the Asian malaria vector, *Anopheles stephensi* (Liston), and a rodent model malaria, *Plasmodium yoelii yoelii*, to investigate whether nutritional quality of larval habitat affects vectorial capacity. We show trans-stadial impacts on a range of traits indicating potential for strong effects of larval rearing condition on subsequent transmission of malaria.

## Methods

### General experimental design

To manipulate larval environment quality, we fed *An. stephensi* larvae differing quantities of food throughout development. Larvae were collected from our standard lab colony at The Pennsylvania State University (this colony was initiated in 2011 with eggs from a longstanding colony maintained at Johns Hopkins University). Newly hatched (<24 h old) first instar larvae were transferred to 265 mL plastic cups containing 80 mL of distilled water at initial densities of 50 larvae per cup. Larvae were maintained on Tetrafin® fish food under standard insectary conditions (26°C ± 0.5°C, 80% humidity, and a 12:12 L:D photoperiod). We exposed larvae to one of two experimental food treatments: 1) a “high” food regime (0.3 mg/individual/day), which is consistent with our standard colony maintenance, and 2) a “low” food regime (0.1 mg/individual/day). These food treatments were selected based on a series of pilot studies. We maintained a constant food environment in each cup by counting larvae and pupae daily, replacing water (kept at a constant volume), and adjusting the amount of food allocated per individual. Each treatment group contained 48 replicate cups. The entire experiment was repeated in time, for a total of two experimental blocks (96 cups per treatment, in all). For mating success and gonotrophic cycle experiments (details below), each larval treatment group contained 15 cups in two experimental blocks (also repeated in time), totaling 30 cups per treatment.

### Quantifying the effects of food treatment on mosquito life history traits

#### Larval survival and development time

To assess effects of experimental food treatment on larval survival, larvae and pupae in each cup were counted daily using an eyedropper. To estimate duration of larval development, and quantify adult mosquito production of each cup, the date of adult female emergence and number of females emerging were recorded.

#### Adult body size

To determine if larval food manipulation generated differences in adult female body size, we took one wing per mosquito (n = 100 per treatment, per experimental block) and mounted wings onto a glass microscope slide using clear nail lacquer. Wings were then measured using a micrometer eyepiece with a standard dissecting microscope. Measurements were taken from the tip of the wing (excluding fringe) to the distal end of the alula. Wing length is known to be positively correlated with body size in mosquitoes [41,42].

#### Gonotrophic cycle, mating success and daily adult survival

To determine effect of larval food treatment on gonotrophic cycle length, females that had emerged 3-5 days prior (and had been housed in mesh cages with males from their respective treatments) were fed to repletion on a Hemotek membrane feeder using pork sausage casing filled with human blood heated to 37°C. Blood-fed females (n = 50 per treatment in each experimental block, for a total of 100 per treatment) were then transferred to individual 50 mL plastic tubes covered with mesh, and provided with a cotton ball moistened with 10% glucose solution that was replenished daily. Each day, sugar moistened cotton balls were removed 4 hours prior to offering a blood meal (also via a Hemotek feeder) for 10 minutes each on four subsequent days. Tubes were monitored daily for eggs, which were counted for each individual female. Mortality was recorded until day 14 after the first blood meal.

Here, gonotrophic cycle is defined as the time (in days) from initial blood meal to laying of the first clutch of eggs, as few individuals laid twice throughout the experiment. Mating success was also assayed by dissection of spermathecae from females in both treatments that never laid eggs throughout the monitoring period. Presence of sperm, whether alive or dead, was considered a successful mating.

Adult survival was monitored for 14 days after the first blood meal, at which point adult females were 17-19 days old. While mosquitoes can live for many weeks under ideal lab conditions [10], this prolonged survival is difficult to interpret as it is generally accepted that few mosquitoes live beyond 2 weeks in field settings [43-45]. Moreover, 14 days after the blood meal is when female mosquitoes are potentially able to transmit malaria and before strong effects of senescence are expected (note the basic vectorial capacity equation is age-independent and assumes a constant rate of mortality in the absence of senescence).

### Quantifying the effects of food treatment on vector competence

In order to assess the effects of larval food treatment on measures of vector competence, we first randomly allocated three-to-five day old individuals from each cup of both treatments across one of four replicate cages (80-100 females per cage). Females were allowed to feed on two anesthetized mice per cage (C57 females, > 6 weeks old) infected with *Plasmodium yoelii yoelii* (clone 17XNL) for thirty minutes. All vertebrate animal work was carried out by trained research technologists under Penn State University IACUC protocols specified in permit #27452. To ensure that mosquitoes received an infectious blood meal at the same adult age, first instar larvae for the low food treatment group were collected from the colony four days earlier than first instars to be allocated to the optimal food treatment group. This was done to adjust for the slower developmental time in the low food treatment group and allow for age-matched comparison of the groups at the same time after administration of the infectious blood meal. Individuals that did not feed were removed from the cage. Infectious feeds were performed at 26°C for optimum host seeking and probing behavior. Immediately following feeding, cages were transferred to a second incubator set at 24°C, as this is the thermal optimum for *Plasmodium yoelii yoelii* growth and replication. After infection, individuals were provided with cotton balls moistened with 10% glucose offered *ad libitum* and replaced daily. Daily survival of infected adults was monitored by counting and removing the dead individuals throughout the duration of the experiment.

Despite our high level of replication at the larval food level (48 cups per treatment per block), we chose to pool individuals from cups into cages (4 per treatment per block). Combining in this way means we cannot trace individual mosquitoes and their resulting infection dynamics back to a specific cup. However, we considered randomization across 4 treatment cages, with use of 2 mice per cage to account for natural mouse-to-mouse variation in gametocytemia, and complete replication through time, sufficient to detect any biologically relevant treatment effects. Putting individual mice on each cup would have increased the number of mice used to unethical levels.

#### Parasite prevalence and intensity

A subset of 25 individuals was taken from each replicate cage at seven days post-infection to assess infection prevalence and intensity. Midguts were dissected in 1X phosphate-buffered saline and examined under a light microscope. Presence or absence of oocysts and the number of established oocysts were quantified. Dissected midguts were saved individually in 1.5 mL tubes and stored in absolute ethanol at -80°C for future genomic DNA analysis (discussed below). At day 16-18 post-infection, 15 individuals were sampled from each replicate cage, and salivary glands were dissected out in 1X phosphate-buffered saline. Glands were examined under a light microscope and scored for presence or absence of sporozoites. To estimate vector competence (*b*, proportion of infectious bites on a susceptible host that lead to an infected host), we used the proportion of mosquitoes with sporozoites disseminated in the salivary glands. This is a standard approximation and assumes that if a mosquito has sporozoites in the salivary glands, it will likely transmit during feeding [3,4,46].

#### Sporozoite replication

To quantify how food treatment affected sporozoite production for each infected mosquito, we performed genomic DNA extraction and qPCR analysis for *Plasmodium* genomes in midguts saved from oocyst dissection. *Plasmodium* genomic DNA was extracted from midguts using the E.Z.N.A. MicroElute Genomic DNA kit (Omega Bio-Tek, as per the manufacturer’s protocol). DNA was eluted in 20 μL of molecular grade water, and the number of parasite genomes present in midguts was quantified using a previously developed qPCR assay [47]. Briefly, reactions were run on an ABI Prism 7500 Sequence Detection System (TaqMan). Initial denaturation was 20 seconds at 95°C followed by 40 cycles of a three-second 95°C denaturation period and a 30-second 60°C period of annealing and extension. Primers and probes were designed to amplify DNA from several *Plasmodium* species. We constructed standard curves for *P. yoelii* genome detection by extracting DNA from a known number of infected mouse red blood cells using the BloodPrep kit (Applied Biosystems) on the ABI Prism 6100 Nucleic Acid Prep Station (as per the manufacturer’s protocol). Parasite production per oocyst was evaluated by dividing the total number of parasite genomes by the number of oocysts quantified for each midgut. We used both sporozoite production per midgut and per oocyst as measures of the efficiency of parasite replication.

### Statistical analyses

All statistical analyses were carried out using IBM SPSS v.21 (Armonk, NY). For all analyses, full factorial models were reduced through backwards elimination of non-significant, higher order interactions, and henceforth non-significant higher order interactions are not reported in our discussion of the results or displayed in our model tables. All models were evaluated for goodness of fit by assessing model deviance per degrees of freedom, log likelihood values and residual plots. To assess significant pairwise comparisons, we used Bonferroni-adjusted post-hoc tests.

We used univariate general linear model (GLM) analysis to determine how food treatment affected adult body size and mating success. Larval and adult survival, day of emergence, number of adults emerged, fecundity and measures of vector competence (oocyst intensity and prevalence, sporozoite prevalence) were analyzed using generalized linear models (GZLM) so that non-normal error distributions could be used in the analysis. All distributions were chosen based on both best model fit and plots of raw data.

Normal distribution was assumed for analysis of day of adult female emergence and gonotrophic cycle. We assumed complimentary log-log, poisson, binomial, negative binomial, and gamma distributions in the analyses for mosquito survival, number of adults emerging and fecundity, oocyst and sporozoite prevalence, oocyst intensity, and number of sporozoites produced for each treatment group, respectively. For each dependent variable in our analyses, food treatment, cage replicate (for parasite traits), and experimental block were included as factors. We included oocyst intensity as a covariate in the model assessing treatment effects on sporozoite production. Across all models concerning characteristics of *Plasmodium* infection, replicate cage was nested within treatment to correct for the fact that mosquitoes in each cage received a blood meal from a different group of mice, and so were not related to one another across treatment groups.

### Quantifying effects of larval food treatment in the context of the vectorial capacity equation

We calculated vectorial capacity using mean parameter estimates quantified from our empirical data (Table 1). In the current study we have no direct measure of the extrinsic incubation period (EIP) of the parasite (*n*), so we assumed the EIP for *P. yoelii* development at 24°C to be 12 days for both treatment groups based on previous research [4]. For daily survival rates (*p*), we used the average rate over the entire 18-day monitoring period. Vector density (*m*) was estimated using the mean of total emerged females per replicate larval cup. We followed convention in using the reciprocal of the mean gonotrophic cycle length as a proxy for daily biting rate (*a*). The proportion of mosquitoes potentially infectious (*b*), was calculated using raw data means for sporozoite prevalence.

**Table 1:** **Output of vectorial capacity equation with experimental parameters**

**Treatment**	**Adult vector density (** ***m*** **)**	**Biting rate (** ***a*** **)**	**Adult daily survival (** ***p*** **)**	**Proportion infectious (** ***b*** **)**	**EIP (** ***n*** **)**	**Vectorial capacity (** ***C*** **)**
*Low diet*	16.7 ± 0.440	0.233 ± 0.009	0.973	0.3 ± 0.042	12	7.155
*High diet*	19.7 ± 0.359	0.293 ± 0.006	0.982	0.3 ± 0.042	12	22.462

All values were calculated using means from our empirical data, except for extrinsic incubation period (EIP), which is assumed based on ideal conditions in previous work. Standard errors of means are displayed for vector density, biting rate, and proportion infectious (sporozoite prevalence).

## Results

### The effects of food treatment on mosquito life history traits

**Figure 1:**
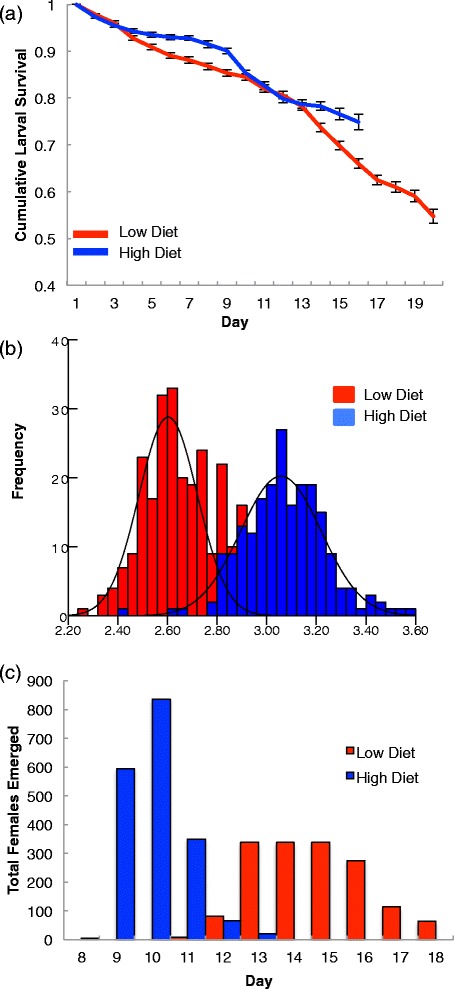
**Effects of larval nutrition on larval development time, adult body size, and day of female emergence. a**. Daily survival from first instar larva to adult. End of survival curve signifies all adults have emerged **b**. Frequency distribution of wing length in females across both treatment groups (red = 0.1 mg/individual/day, n = 213, blue = 0.3 mg/individual/day, n = 206). Groups are significantly different from each other (treatment, p = <0.001, univariate GLM). **b**. Daily survival from first instar larva to adult. End of survival curve signifies all adults have emerged. **c**. Female emergence per day; depicts total adults (all replicates, all blocks) emerged at each day after hatching. Female emergence in the low diet is characterized by fewer individuals, as well as a more extended pattern of emergence with no distinct peak.

Food treatment significantly affected larval survival (and henceforth the number of emerging adult females). Larvae reared on low food diets did not survive as well as those reared on high food diets (Figure 1a). However, this effect was strongly mediated by day (analyzed here as a covariate) and interactions among food treatment and experimental block and experimental block and day (Table 2, GZLM, interval-censored survival analysis). Although the trend of reduced larval survival and subsequent adult emergence in the low food group remained consistent across experimental blocks, the first block showed an initial (days 2-5 after hatching) sharp decline in the survival of the high food group, which did not replicate in the second experimental block resulting in the significant *treatment x day x block* interaction (Table 2). We did see significant replicate effects; however, upon plotting Kaplan-Meier survival curves for each of the 48 replicates per treatment per block, these effects were due to several cups that exhibited unusually high or low larval mortality (data not shown). However, the trends remain the same and when assessing the final productivity of each cup (total number of females), replicate is not a significant effect.

**Table 2:** **Generalized linear model output for mosquito life history traits**

	***Larval survival*** **(n = 3177)**			***Vector density*** **(n = 190)**			***Adult survival larval survival*** **(n = 414)**			***Gonotrophic cycle*** **(n = 145)**		
**Factors**	**Wald** ***X2***	**d.f.**	**p - value**	**Wald** ***X2***	**d.f.**	**p - value**	**Wald** ***X2***	**d.f.**	**p - value**	**Wald** ***X2***	**d.f.**	**p - value**
*Intercept*	**a**	**a**	**a**	**7817.07**	**1**	**<0.001**	**122.43**	**1**	**<0.001**	**2713.148**	**1**	**<0.001**
*Treatment*	**78.06**	**1**	**<0.001**	**114.46**	**1**	**<0.001**	**11.63**	1	**<0.001**	**35.687**	**1**	**<0.001**
*Replicate *(*treatment*)	768.72	94	<0.001	99.03	94	**<0.001**	4.36	6	0.629	N.A.	N.A.	N.A.
*Block*	**61.12**	**1**	**<0.001**	**32.69**	**1**	0.341	**18.34**	**1**	**<0.001**	**9.895**	**1**	**0.002**
*Day*	**1815.62**	**1**	**<0.001**	N.A	N.A	N.A	**355.22**	**1**	**<0.001**	N.A.	N.A.	N.A.
*Treatment x block*	**50.545**	**1**	**<0.001**	N.S	N.S	N.S	N.S	N.S	N.S	N.S.	N.S.	N.S.
*Treatment x day*	0.334	**1**	0.563	N.A	N.A	N.A	N.S	N.S	N.S	N.A.	N.A.	N.A.
*Block x day*	**27.485**	**1**	**<0.001**	N.A	N.A	N.A	N.S	N.S	N.S	N.A.	N.A.	N.A.
*Treatment x block x day*	**32.537**	**1**	**<0.001**	N.S	N.S	N.S	N.S	N.S	N.S	N.A.	N.A.1	N.A.

Bold indicates significance at α = 0.05. (Model fit assessed by value of deviance per degrees of freedom: larval survival = 1.450, vector density = 0.934, adult survival = 8.967, gonotrophic cycle = 0.765). P-values are reported only for significant interactions and first order terms. Table reflects the final output of a backwards-eliminated full factorial model. N.S. = not significtant, N.A. = not applicable, a = SPSS unable to compute due to numerical issues.

Low food environments produced, on average, significantly fewer total adult females per replicate cup than the high food environments (Figure 1c). In the low diet, mean emergence was 16.7 females (S.E. = 0.44), while high diet cups produced a mean of 19.7 (S.E. = 0.359) (p = <0.001, GZLM, Table 2). Adult females emerging from the high food treatment group had significantly larger wings than those emerging from the low food treatment group, with little overlap between the two groups (Figure 1b).

**Figure 2:**
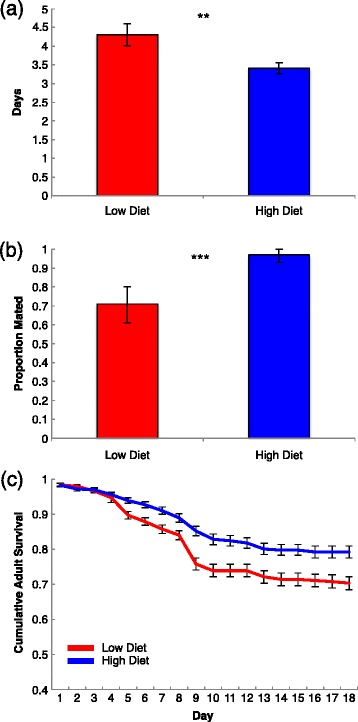
**Effects of larval nutrition on adult female reproductive traits and post**-**infectious survival. a**. Length of first gonotrophic cycle, used to estimate biting rate. Treatments are significantly different from one another (p = <0.001, univariate GLM). Bars represent 95% confidence intervals around mean values. Low diet n = 55, high diet n = 90. **b**. Mean proportion of individuals with sperm present in spermathecae. Individuals from optimal food treatments exhibited higher mating success than those from low food treatments. Bars are significantly different from one another (p = <0.001). Error bars represent the 95% confidence intervals around the mean values. Low diet n = 85, high diet n = 93. **c**. Daily post infectious adult female survival of each group. Note the most significant changes occur between day four and ten.

Length of gonotrophic cycle was significantly shaped by larval food treatment (Table 2), and females from low larval food diets had gonotrophic cycles that were on average one day longer than mosquitoes fed high food diets (Figure 2a). There was a significant effect of experimental block, with individuals in the second taking slightly longer to lay the first clutch of eggs, but this effect was consistent across treatments, resulting in no significant *treatment x block* interactions. Larval food treatment also significantly affected mating success (presence of sperm in spermatheca; Figure 2b) and fecundity. Females from the low food treatment were less likely to be mated than those from the high food treatment (mean proportion mated = 0.71 ± .45, 0.97 ± 0.18, respectively), and those in the low food treatment that did lay eggs, laid significantly fewer than high food females (mean size of first clutch = 28.67 ± 2.011, 64.14 ± 1.797, respectively; treatment p <0.001, GZLM, Poisson distribution).

### Effects of food treatment on vector competence

Larval food treatment significantly affected adult infected female survival (p = 0.001, GZLM, interval-censored survival, Table 2). The difference was not significant until day five post-infection, when survival in the low food group fell sharply, then leveled off at day 10, with little mortality in either group for the remainder of the observation period (Figure 1c).

**Figure 3:**
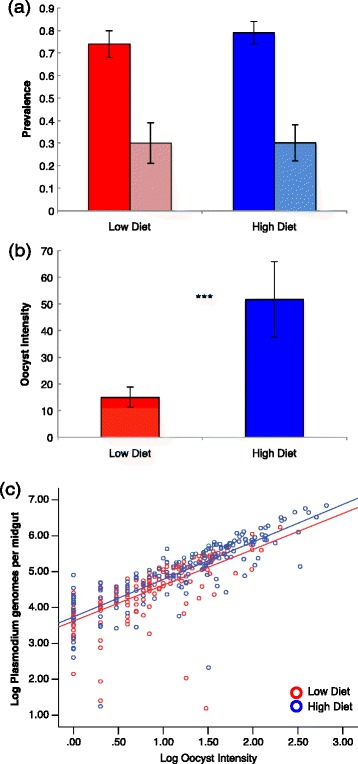
**Effects of larval nutrition on parasite dynamics in midgut (oocyst) and salivary gland (sporozoite) stages. a**. Prevalence of oocysts (dark-colored bars) and sporozoites (light-colored bars) in both treatments. There is no significant difference between low and high food treatment individuals for oocysts (p = 0.164, GZLM) or sporozoites (p = 0.944, GZLM). Error bars represent the 95% confidence intervals for mean values. **b**. Infection load for each treatment as quantified by oocyst intensity in infected individuals. Treatments are significantly different from one another (p < 0.001, GZLM). Error bars represent the 95% confidence intervals for mean values. **c**. Relationship between oocyst intensity and sporozoite production (*Plasmodium* genomes per midgut). Both oocyst intensity and total genomes are log-transformed for clarity in visualization. Treatments are significantly different from one another (p < 0.001, GZLM).

Despite differences in adult survivorship, food treatment had no significant effect on either oocyst or sporozoite prevalence (Table 3, Figure 3a). In contrast, food treatment significantly influenced oocyst intensity (Table 3, Figure 3b), and females from the low food group had an overall lower oocyst burden than those from the high larval food group. This effect was consistent across experimental blocks, despite the difference in infection levels between experiments.

**Table 3:** **Generalized linear model output for vector competence and parasite dynamics**

	***Oocyst prevalence*** **(n = 417)**			***Sporozoite prevalence*** **(n = 239)**			***Oocyst intensity*** **(n = 316)**			***Plasmodium genomes per midgut*** **(n = 316)**		
**Factors**	**Wald** ***X2***	**d.f.**	**p - value**	**Wald** ***X2***	**d.f.**	**p - value**	**Wald** ***X2***	**d.f.**	**p - value**	**Wald** ***X2***	**d.f.**	**p - value**
*Intercept*	**104.07**	**1**	**<0.001**	**35.91**	**1**	**<0.001**	**2535.06**	**1**	**<0.001**	**14425.51**	1	**<0.001**
*Treatment*	1.93	1	0.164	0.01	1	0.944	**76.61**	**1**	**<0.001**	**43.42**	1	**<0.001**
*Replicate*(*treatment*)	5.57	6	0.473	9.04	6	0.171	**20.79**	6	**0.002**	2.20	6	0.900
*Block*	**35.91**	**1**	**<0.001**	**12.20**	**1**	**<0.001**	**79.3**	**1**	**<0.001**	**74.68**	1	**<0.001**
*Intensity*	N.A.	N.A.	N.A.	N.A.	N.A.	N.A.	N.A.	**N.A.**	N.A.	**114.83**	1	**<0.001**
*Treatment x block*	N.S.	N.S.	N.S.	N.S.	N.S.	N.S.	**33.79**	**7**	**<0.001**	**7.18**	1	**<0.001**
*Treatment x intensity*	N.A.	N.A.	N.A.	N.A.	N.A.	N.A.	N.A.	N.A.	N.A.	**16.15**	1	**<0.001**
*Block x intensity*	N.A.	N.A.	N.A.	N.A.	N.A.	N.A.	N.A.	N.A.	N.A.	**35.96**	1	**<0.001**

Bold indicates significance at α = 0.05. (Model fit assessed by value of deviance per degrees of freedom: oocyst prevalence = 1.379, sporozoite prevalence = 1.748, oocyst intensity = 1.422, genomes per midgut = 1.286).

P-values are reported only for significant interactions and first order terms. Table reflects the final output of a backwards-eliminated full factorial model. Intensity is included as a covariate only for *Plasmodium* genomes, as it would be an inappropriate variable for all other parasite traits reported in this table. N.S. = not significtant, N.A. = not applicable.

Individuals fed a high food diet exhibited a greater number of parasite genomes per oocyst than mosquitoes fed a low food diet (mean of 1.69 × 10^5^ and 2.2 × 10^5^ genomes per midgut for low and high treatment, respectively) (Table 3). Unsurprisingly, the number of *Plasmodium* genomes per midgut increased with oocyst intensity. However, the slope of the positive relationship between oocyst intensity and number of *Plasmodium* genomes is steeper for individuals in the high food treatment (slope = 9.38E3, R^2^ = 0.787) than for the low food treatment (slope = 6.44E3, R^2^ = 0.747), as indicated by the significant interaction between food treatment and oocyst intensity (*treatment x oocyst intensity*, Table 3).

Here, the significance of experimental block is likely due to the fact that oocyst intensity in the second block (mean = 13.26) was lower than that of the first block (mean = 46.36). Though the trends replicate and remain significant, the magnitude of the relationship between intensity and genome count is much greater in the first block.

### Effects of food treatment on vectorial capacity

For daily survival rates (*p*), we took the average rate over the entire 18-day monitoring period, which resulted in *p* = 0.973 and *p* = 0.983 for the low and high food groups, respectively. Mean adult female density (*m*) per replicate cup in the low diet was 16.7 females (S.E. = 0.44), while high diet cups produced a mean of 19.7 (S.E. = 0.359) (p = <0.001, GZLM, Table 2). Gonotrophic cycle for low food individuals was 4.29 days (the reciprocal of which gives an average biting rate per day of *a* = 0.233, S.E. = .009), while high food individuals had an average cycle length of 3.41 days (*a* = 0.293, S.E. = 0.006). Sporozoite prevalence (*b*) mean was 0.30 for both groups (S.E. = 0.042). These parameter values yielded mean vectorial capacities of C = 7.155 for mosquitoes from the low food environment and C = 22.462 for those from the high food environment.

## Discussion

In this study we demonstrate effects of variation in food abundance at the larval stage on both larval survival and a suite of adult mosquito life history traits that combine to determine vectorial capacity. The treatment effects equate to a *c*.70% relative reduction in malaria transmission potential for mosquitoes from the low food environment, or conversely, a relative increase in transmission potential of *c*.310% for mosquitoes from the high food environment.

The most obvious influence of larval food treatment was the impact on daily survival and prolonged development of larvae in poor nutritive environments. Longer development times and smaller proportion of mosquitoes successfully pupating and eclosing, has been reported in *Anopheles gambiae*, *Aedes triseriatus* and *Anopheles darlingi* [14,48,49]. Further, although larval development is not a direct component of vectorial capacity, it will affect vector population growth rates and possibly vector density (depending on the nature of the density dependence [50]). Adult survival has also been shown to be affected by quality of larval habitat in other mosquitoes [51,52]. One recent study suggested that quality of larval habitat had no effect on subsequent survival of adult *An. stephensi* and hence, no implications for transmission [39]. However, this study monitored adult survival for 5 days only, which is much shorter than the incubation period of the parasite and so provides little insight into ultimate impacts on the number of adults potentially able to transmit the parasite (note that differences in survival between treatments in our study only emerged between days 5-9).

Larval food treatment also carried over to have a significant effect on adult body size. Several studies have shown positive correlation between body size and fitness in vector species. For example, in *Aedes albopictus* reared in both the laboratory and the field, pupal mass and wing length were consistently correlated positively with fecundity [53]. In field-collected *Aedes communis*, wing length was a significant predictor of longevity when the adult females were food stressed [54]. Studies on *Anopheles gambiae* show that reduction in resource availability through increasing larval density significantly decreases adult mass in males and females. Reduced body mass was demonstrated to be negatively correlated with both age at pupation and mating success, suggesting that both development time and body size have an influence on adult fitness [55-57].

Larval food treatment also impacted duration of the gonotophic cycle, and hence the reciprocal estimate of daily biting rate. Changes in gonotrophic cycle length were relatively small (<1 day) but because biting rate enters into the vectorial capacity equation as a squared term (reflecting that one bite is required for a mosquito to acquire the parasite and at least one other required to pass it on), even small changes can contribute to differences in overall vectorial capacity [18,58,59]. The prolonged gonotrophic cycle length we observe in small females could be due to decreased nutritional reserves (as could the effects on fecundity). This reduction in reserves has been shown in *Aedes aegypti* females to prolong especially the first gonotrophic cycle, as more than 50% of the lipid resources needed to develop eggs are from larval stores [33]. In nature, the timing of the initial blood meal could also be delayed if a smaller proportion of females engage in blood feeding, as seen in *Anopheles darlingi* emerging from poor larval habitats [40], or if smaller females have higher initial preference for sugar feeding as opposed to blood feeding [60]. These latter effects would potentially further impact vectorial capacity.

Differences in fecundity and mating success, while they do not contribute directly to vectorial capacity, could further shape vector density. It has been demonstrated in several studies that fecundity is positively correlated with body size in several anopheline species, due to both available teneral reserves for ovarian development as well as larger blood meal size taken by larger females [61-63]. Possible explanations for impacts on mating success include depressed mate-seeking in small individuals of both sexes due to energetic demand [64,65]. *Anopheles gambiae* males have been demonstrated to preferentially select larger females for mates [66], so low food females may have not presented as attractive mates for their conspecific males. Additionally, differences between treatments could affect the viability and number of sperm [67]. In our studies, mosquitoes were restricted to mating within their respective treatment groups. These trends in mating success might change if mosquitoes were allowed to mate freely with individuals of differing sizes from diverse larval habitats [68].

The effects of larval diet on parasite development and overall vector competence were mixed. We observed differences in parasite intensity and genomic DNA replication, suggesting that larger mosquitoes might offer greater nutritional resources for parasite exploitation within the insect, but found no effect on parasite prevalence at either the oocyst or sporozoite stage. We used a rodent malaria as an established model [69] but there are recognized differences between typical infection intensities of rodent and human malarias [70], and the resultant mosquito immune responses [71,72]. We also acknowledge that we are using a laboratory strain of *An. stephensi* that has known susceptibility to certain *Plasmodium* species and that natural sympatric vector-parasite pairings can differ in baseline competence [73]. The functional importance of these differences is slightly unclear, but they complicate translation of results across systems and we do not suggest that our calculations of vectorial capacity can be applied directly to the field. That said, human malaria parasites do require mosquito resources for development [74-77] so there is no reason to think that host condition is unimportant. Several previous studies have demonstrated effects of larval density on adult traits including body size [15,55,56], survival [52], blood intake, and mating competitiveness [78,79] suggesting our results to be robust. Comparative differences might be enhanced or diminished in the face of the complexity that exists in the field.

The effects we observe on parasite replication rate suggest the additional intriguing possibility that host condition could affect the extrinsic incubation period (EIP) of the parasite. We did not measure EIP in our study as we dissected salivary glands at a single time point during infection. However, differences in parasite replication rate due to the influence of temperature, for example, are known to affect length of the EIP, with important consequences for vectorial capacity [4,10] We are not aware of any studies exploring the effects of mosquito condition (larval through to adult) on malaria parasite development but nutritionally-dependent parasite replication has been observed in other invertebrate-parasite systems [30,80-82]. If EIP is affected by mosquito condition, this could add to the influence of larval habitat on malaria transmission.

Russell *et al*. (47) analysed a time series of *An. gambiae* abundance and condition (body size) data collected in a high transmission setting in central Tanzania. Their study revealed marked variation in adult body size across the year linked to rainfall-driven changes in availability (and likely quality) of larval breeding habitats. The density dependent feedbacks between mosquito condition and carrying capacity of larval habitats provided an elegant explanation for the observed adult mosquito population dynamics. Our data indicate that variation in quality of larval habitats could have important impacts on vectorial capacity beyond predicted effects on mosquito abundance.

## Conclusions

Caveats regarding the laboratory nature of our experimental system notwithstanding, the magnitude of the effects we observe are non-trivial. A 70% reduction in vectorial capacity is of the same order as reported from well implemented control programs using insecticide sprays or insecticide treated nets [83-86]. Thus, variation in larval habitat quality could have a marked influence on the temporal and spatial dynamics of malaria transmission. Larval resource effects could also have important implications for control. For example, poorly implemented larval control, such as inefficient larviciding, could possibly alleviate density effects, increasing resource availability for the remaining larvae. This reduced larval competition might lead to inadvertent increases in transmission potential. Conversely, reduced nutrition via increases in larval competition could reduce transmission potential and might provide a partial explanation for the so-called ‘paddy paradox’, whereby malaria burden remains low in rice irrigation areas in spite of very high mosquito densities [87]. The potential for such effects reinforces the need for better understanding the effects of environmental quality on larval and adult mosquito ecology [88].
